# Concomitant Loss of p120-Catenin and β-Catenin Membrane Expression and Oral Carcinoma Progression with E-Cadherin Reduction

**DOI:** 10.1371/journal.pone.0069777

**Published:** 2013-08-06

**Authors:** Kazunobu Sasaya, Haruka Sudo, Genta Maeda, Shuichi Kawashiri, Kazushi Imai

**Affiliations:** 1 Department of Biochemistry, School of Life Dentistry at Tokyo, The Nippon Dental University, Tokyo, Japan; 2 Department of Oral Surgery, School of Medicine, Kanazawa University, Kanazawa, Japan; Wayne State University School of Medicine, United States of America

## Abstract

The binding of p120-catenin and β-catenin to the cytoplasmic domain of E-cadherin establishes epithelial cell-cell adhesion. Reduction and loss of catenin expression degrades E-cadherin-mediated carcinoma cell-cell adhesion and causes carcinomas to progress into aggressive states. Since both catenins are differentially regulated and play distinct roles when they dissociate from E-cadherin, evaluation of their expression, subcellular localization and the correlation with E-cadherin expression are important subjects. However, the same analyses are not readily performed on squamous cell carcinomas in which E-cadherin expression determines the disease progression. In the present study, we examined expression and subcellular localization of p120-catenin and β-catenin in oral carcinomas (*n* = 67) and its implications in the carcinoma progression and E-cadherin expression using immunohitochemistry. At the invasive front, catenin-membrane-positive carcinoma cells were decreased in the dedifferentiated (p120-catenin, *P* < 0.05; β-catenin, *P* < 0.05) and invasive carcinomas (p120-catenin, *P* < 0.01; β-catenin, *P* < 0.05) and with the E-cadherin staining (p120-catenin, *P* < 0.01; β-catenin, *P* < 0.01). Carcinoma cells with β-catenin cytoplasmic and/or nuclear staining were increased at the invasive front compared to the center of tumors (*P* < 0.01). Although the p120-catenin isoform shift from three to one associates with carcinoma progression, it was not observed after TGF-β, EGF or TNF-α treatments. The total amount of p120-catenin expression was decreased upon co-treatment of TGF-β with EGF or TNF-α. The above data indicate that catenin membrane staining is a primary determinant for E-cadherin-mediated cell-cell adhesion and progression of oral carcinomas. Furthermore, it suggests that loss of p120-catenin expression and cytoplasmic localization of β-catenin fine-tune the carcinoma progression.

## Introduction

Oral squamous cell carcinoma is a common malignant neoplasm of the head and neck and prevalence is predicted to increase in the next few decades. Regardless of therapeutic approach, location, or stage of the disease, more than 50% of patients experience a relapse [1]. Although treatment failure can be attributed to multiple factors, understanding molecular the mechanisms regulating carcinoma progression will aid in developing novel strategies for cancer therapy. At the first step of progression, carcinoma cells must sequester from their primary sites and invade into the basement membrane and underlying tissues. Here, they expose themselves to a more advanced state of progression as they weaken cell-cell adhesion and invade in the form of small subsets or individual cells. During the invasion, interaction with the microenvironments surrounding the carcinoma cells at the invasive front play an important role in progression [2].

Epithelial cells develop tight cell-cell adhesions as compared to other cell-types. They form distinct tissue layers through the formation of tissue boundaries and change tissue shapes causing cell rearrangements or the conversion between histological cell states and the long-range migration of cells. The stratified squamous epithelium that covers the oral cavity is especially well organized and form strong cell-cell adhesions mediated by cadherins. Cadherins are a superfamily of calcium-dependent transmembrane proteins that are developmentally regulated and evolutionally conserved cell-cell adhesion molecules. Within the cadherin superfamily, epithelial-type E-cadherin plays a decisive role for development and maintenance of epithelium at the adherence junction [3].

The cytoplasmic domain of E-cadherin binds with catenins. Catenins mediate the interaction of E-cadherin with the cytoskeleton including actin filaments and microtubules, and stabilize adherence junctions [4]. p120-catenin binds to the juxtaposed cell membrane region of the cytoplasmic domain and regulates microtubule accumulation at the junctions [5]. Exposure of the p120-catenin binding region initiates E-cadheirn endocytosis and destabilizes the junctions [6,7], while loss of p120-catenin expression enhances cellular migration [8]. Multiple p120-catenin isoforms (isoform 1-4) are generated by alternative usage of the translation start sites. The isoforms preserve the armadillo-repeats domain, which binds to the juxtaposed region of the cell membrane, but vary in the length of the NH_2_-terminal region; isoform 1 is the longest variant and isoform 4 is the shortest variants [9].

β-catenin binds to the COOH-terminal of the E-cadherin cytoplasmic domain through the armadillo-repeats domain and mediates ligation of actin filaments [10]. The cytoplasmic pool of β-catenin, free of E-cadherin, is increased by WNT signaling. β-catenin translocates into the nucleus and regulates the WNT target gene transcription, resulting in enhancement of migration, proliferation, invasion and metastasis of carcinoma cells [11]. This indicates that both catenins primarilly control the intercellular adhesion of carcinoma cells, but play dynamically different roles when they dissociate from E-cadherin.

Both catenins can also support neural N-cadherin-mediated cell-cell adhesion as in E-cadherin, and E-cadherin to N-cadherin switch, enhancing carcinoma progression [2,3]. However, previous studies reported that catenin expression declines with loss of E-cadherin expression in adenocarcinomas regardless of the gain of N-cadherin expression [12–14]. Thus underlies the difficulty in concluding the role of catenin in carcinoma progression. Oral carcinomas advance their pathological states with the loss of E-cadherin expression and negligibly express N-cadherin [15], indicating that loss of E-cadherin is a critical determinant for the progression. This suggests that expression and subcellular localization of catenins differentially regulate oral carcinoma progression. The present study intends to examine the immunohistochemical expression and subcellular localization of p120-catenin and β-catenin in oral carcinomas, and consider the association with E-cadherin expression and the clinicopathological parameters.

## Results

### Catenin expression in carcinoma cell lines

**Figure 1:**
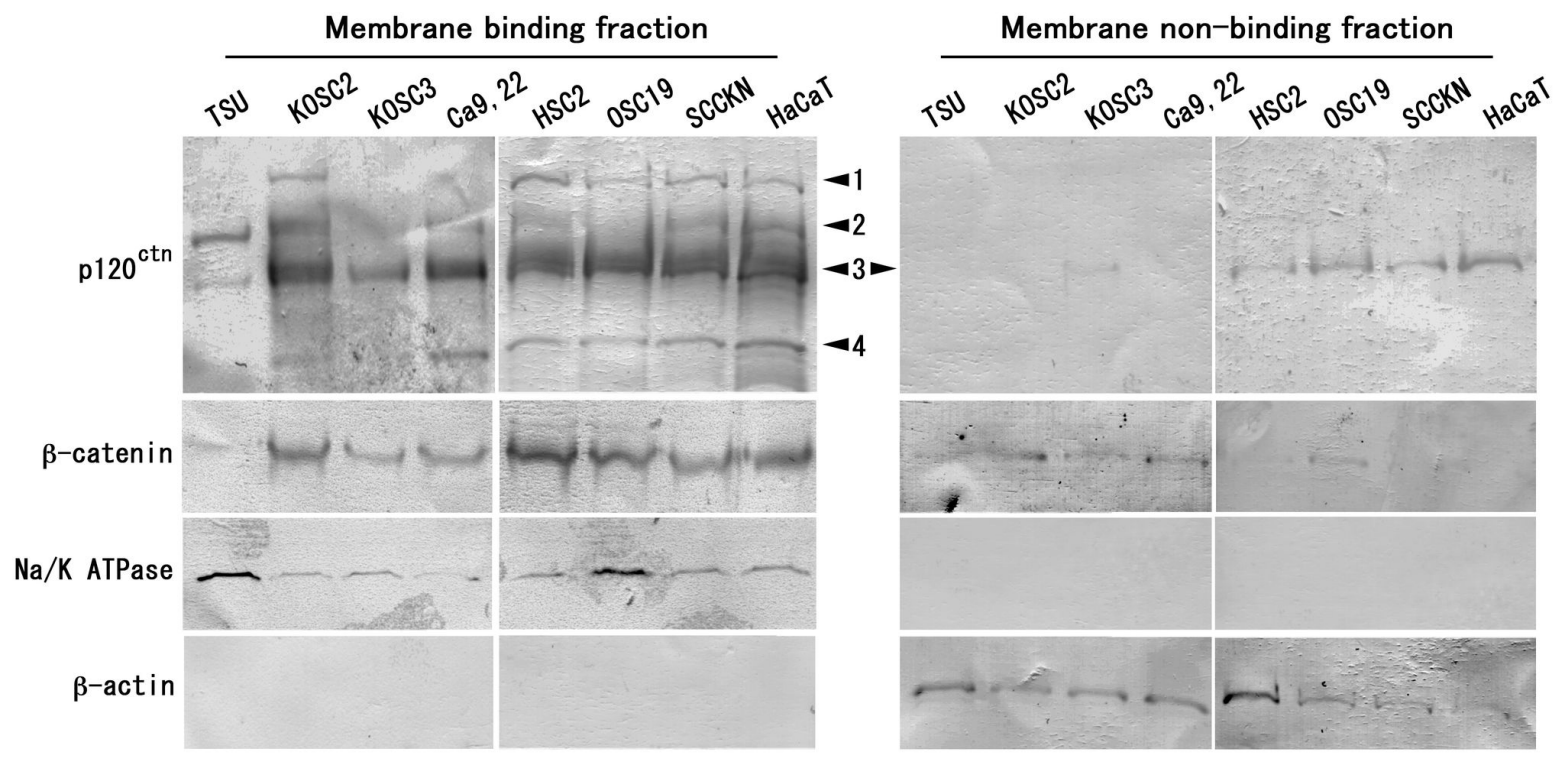
Expression of p120-catenin and β-catenin in oral carcinoma cells and normal keratinocytes. Membrane binding fraction and membrane non-binding fraction of oral carcinoma cells (TSU, KOSC2, KOSC3, Ca9.22, HSC2, OSC19, SCCKN) and normal keratinocytes (HaCaT) were subjected to the immunoblot for p120-catenin (isoform 1-4) and β-catenin. Na/K ATPase and β-actin were probed as the control of membrane binding protein and membrane non-binding protein, respectively.

Since p120-catenin protein consists of four isoforms, we used an antibody that can equally recognize all of them [16]. All isoforms (isoform 1, 120 kDa; isoform 2, 110 kDa; isoform 3, 97 kDa; isoform 4, 75 kDa) were detected in the membrane-binding (MB) fraction, and isoform 3 was predominant in normal keratinocytes (HaCaT cells) and oral carcinoma cells. Regardless of the isoform, expression levels in carcinoma cells were frequently below HaCaT cells (Figure 1). β-catenin was detected as a single band of 92 kDa. Although the biological role is uncertain, p120-catenin isoform 3 and β-catenin in the membrane-non-binding (MNB) fractions tended to be expressed in a different set of cells.

### Expression of catenin in the normal oral epithelium

**Figure 2:**
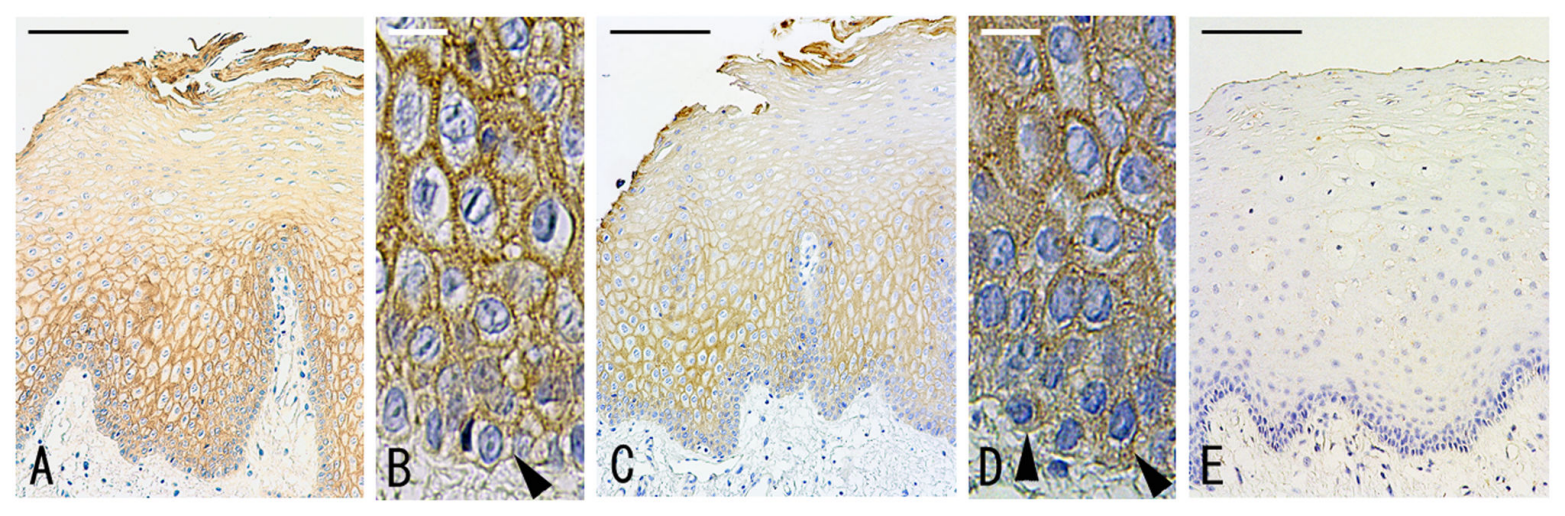
Localization of p120-catenin and β-catenin in oral normal epithelium. Expression and localization of p120-catenin (A, B) and β-catenin (D, E) in oral normal epithelium was examined by immunostaining. Arrowheads indicate the basal cells. D: negative control. Bar = 65 µm (A, C, E), and 4.3 µm (B, D).

Both catenins immunolocalized at the cell membrane region of normal oral epithelial cells from the basal to suprabasal layers and weaken the reactivity toward the cornified layer (Figure 2). At the basal cells, p120-catenin staining was weak and restricted to the cell membrane, whereas β-catenin diffusely localized at the cytoplasm in a high frequency. None of stromal cells were stained with these catenins.

### Expression of p120-catenin in oral carcinomas

**Figure 3:**
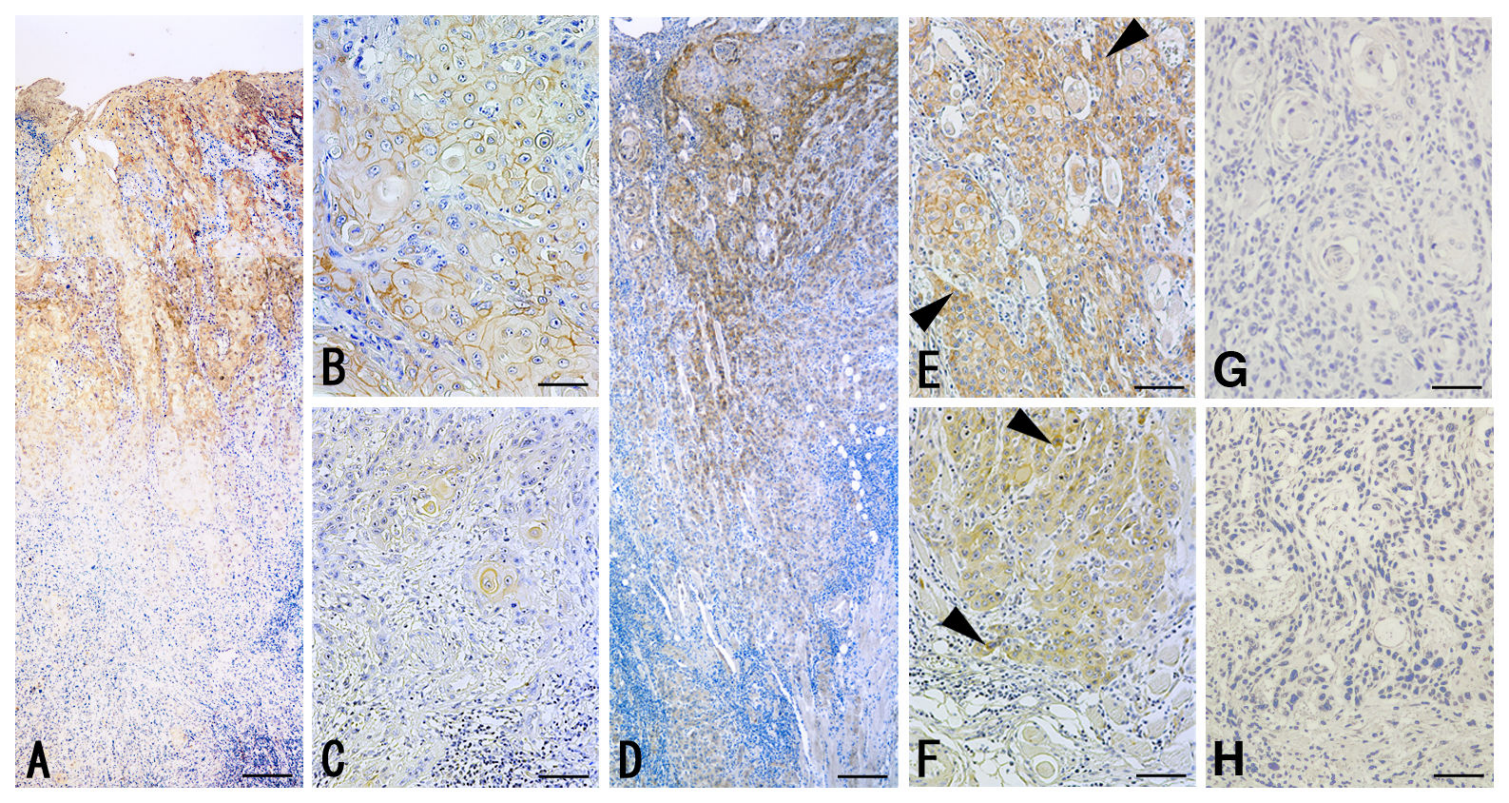
Localization of p120-catenin and β-catenin in oral carcinomas. Expression and localization of p120-catenin (A–C) and β-catenin (D–F) in oral carcinomas were examined by immunostaining. The staining at the center of tumor (B, E, G) and the invasive front (C, F, H) was showed in high-power view. Non-immune IgG was use as a negative control instead of primary antibodies (G, H). Arrowheads indicate the cytoplasmic staining of β-catenin in D and the nuclear staining in F. Bar = 80 µm (A, D), and 15 µm (B, C, E–H).

The p120-catenin was strongly detected at the center of the tumors but rapidly decreased toward the invasive front (Figure 3A–C). Since catenins have distinct roles at the cell membrane and in the cytoplasm [11,17], we independently calculated the percentage of membrane-positive and cytoplasm-positive carcinoma cells at the center and invasive front of the tumors. Although the percentage of membrane-positive cells was significantly reduced at the invasive front (center, 67.4 ± 26.7%, mean ± S.D.; invasive front, 24.8 ± 30.1%; *P* < 0.01), there was no statistical significance between the cytoplasm-positive cells at the center (18.7 ± 23.1%) and the invasive front (19.1 ± 25.6%; *P* = 0.91). The percentage of membrane-positive cells inversely correlated with the cytoplasm-positive cells at the center (*P* < 0.01, R^2^ = 0.13) but not at the invasive front (*P* = 0.90, R^2^ = 0.00). The nuclear staining was not detected in this study.

The percentage of membrane-positive cells was decreased in parallel with tumor dedifferentiation (center, *P* < 0.01; invasive front, *P* = 0.01; Table 1). At the invasive front, the membrane-positive cells were declined in a group of high-invasive carcinomas (grades 1 and 2 vs. 4C and 4D, P < 0.01; Table 1; see Table S1 for other parameters). The statistical analyses for the cytoplasmic staining were summarized in Table S2.

**Table 1:** Membrane immunoreactivities of p120-catenin and β-catenin and clinicopathological implications.

**p120-catenin**					
**Category**	**Subcategory**	**Center**		**Invasive front**	
		Mean ± SD	*P* ^†^	Mean ± SD	*P* ^†^
Differentiation	Well	76.20 ± 17.80	<0.01	36.07 ± 31.78	0.01
	Moderate	73.33 ± 20.75		19.75 ± 25.70	
	Poor	36.00 ± 31.75		8.15 ± 24.68	
Invasion^*^	Grade 1	76.70 ± 17.04	0.11	47.30 ± 33.49	<0.01
	Grade 2	78.00 ± 24.86		21.67 ± 28.93	
	Grade 3	69.79 ± 21.76		27.75 ± 7.75	
	Grade 4C	59.38 ± 38.72		7.75 ± 17.29	
	Grade 4D	35.71 ± 27.40		0.00 ± 0.00	
**β-catenin**					
**Category**	**Subcategory**	**Center**		**Invasive front**	
		Mean ± SD	*P* ^†^	Mean ± SD	*P* ^†^
Differentiation	Well	64.30 ± 26.65	0.01	22.13 ± 21.07	0.01
	Moderate	76.58 ± 20.70		32.63 ± 30.40	
	Poor	44.46 ± 26.65		9.00 ± 16.07	
Invasion^*^	Grade 1	71.50 ± 14.10	0.91	23.80 ± 22.95	0.03
	Grade 2	68.17 ± 23.61		26.25 ± 21.47	
	Grade 3	63.46 ± 33.26		27.75 ± 27.54	
	Grade 4C	61.00 ± 35.86		16.88 ± 28.36	
	Grade 4D	64.29 ± 23.64		4.42 ± 5.71	

* Patients were categorized by mode of invasion by Yamamoto et al. (1983).

† Welch’s ANOVA

### Expression of β-catenin in oral SCCs

**Figure 4:**
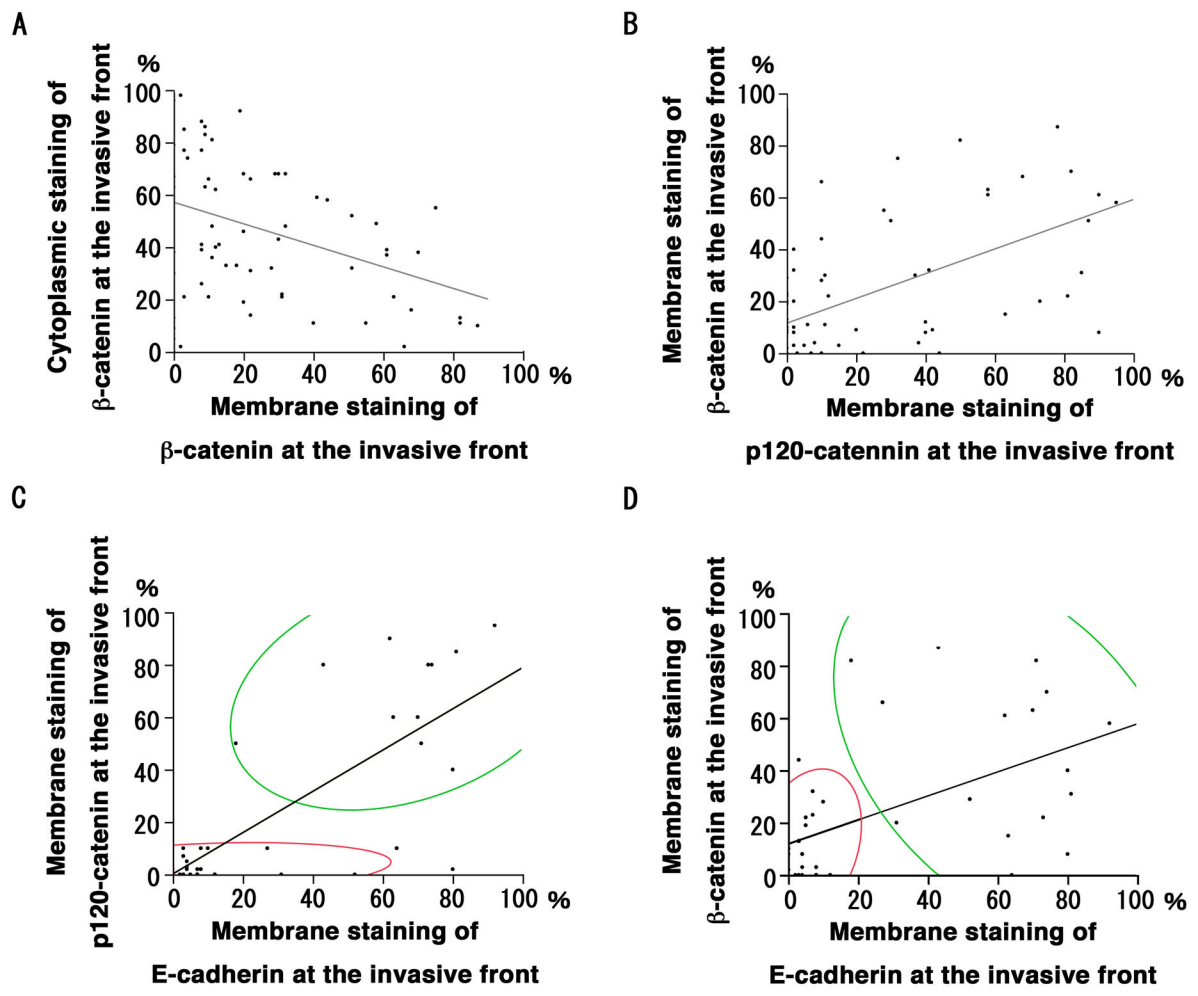
Correlation of percentage of catenin- and E-cadherin-positive carcinoma cells. A: The percentage of β-catenin-membrane-positive and –cytoplasm-positive at the invasive front were inversely correlated (*P* < 0.01, R^2^ = 0.11). B: The positive correlation of the percentage of membrane-positive carcinoma cells for p120-catenin and β-catenin at the invasive front (*P* < 0.01, R^2^ = 0.33). C: The percentage of membrane-positive carcinoma cells for p120-catenin and E-cadherin at the invasive front showed the positive correlation (*P* < 0.01, R^2^ = 0.60). Cluster analysis grouped carcinomas into p120^high^/E-cadherin^high^ (green) and p120^low^/E-cadherin^low^ (red) fractions. D: The positive correlation between the percentage of membrane-positive carcinoma cells for β-catenin and E-cadherin at the invasive front (*P* < 0.01, R^2^ = 0.29) was observed. Carcinoma cells were categorized into β-catenin^high^/E-cadherin^high^ (green) and β-catenin^low^/E-cadherin^low^ (red) fractions.

β-catenin membrane staining was observed in most carcinoma cells at the center (Figure 3D and 3E). Carcinoma cells rapidly declined with respect to membrane staining at the invasive front but showed increased cytoplasmic staining and nuclear staining (Figure 3D and 3F). The percentage of membrane-positive cells at the invasive front (23.3 ± 31.2%) was significantly lower than at center (64.9 ± 28.6%, *P* < 0.01), and the cytoplasm/nucleus-positive cells at the invasive front (47.5 ± 31.2%) were higher than that at center (23.9 ± 25.4%, *P* < 0.01). An inverse correlation between membrane-positive cells and cytoplasm/nucleus-positive cells at the center (*P* < 0.01, R^2^ = 0.39) and invasive front (*P* < 0.01, R^2^ = 0.11; Figure 4A) was found. The membrane staining was decreased with tumor dedifferentiation (center, *P* = 0.01; invasive front, *P* = 0.01; Table 1; see Table S3 in detail). Membrane staining also decreased in parallel with the mode of invasion at the invasive front (*P* = 0.03, Table 1). The cytoplasmic/nuclear staining and its correlation with the clinicopathological parameters are summarized in Table S4.

### Correlation between expression of catenins and E-cadherin

At the invasive front, membrane staining of p120-catenin was positively correlated with that of β-catenin (Figure 4B). The percentage of E-cadherin-positive carcinoma cells rapidly decreases at the invasive front [15], suggesting the close association of E-cadherin and catenin expression. To this end, the percentage of E-cadherin-positive cells was quoted from our recent study [15] and compared with that of catenin-positive cells (Table 2, see Table S5 for detail). Percentage of E-cadherin positive cells at the membrane was correlated with the p120-catenin (Figure 4C) and β-catenin positive cells (Figure 4D). The cluster analysis divided carcinoma cells into p120-catenin^high^/E-cadherin^high^ and p120-catenin^low^/E-cadherin^low^ groups (*P* < 0.01, R^2^ = 0.60, Figure 4C). The analysis on β-catenin and E-cadherin categorized a β-catenin^low^/E-cadherin^low^ group and another (*P* < 0.01, R^2^ = 0.29, Figure 4D).

**Table 2:** Correlation of percentage of catenin and cadherin positive carcinoma cells.

**Center**			
Parameter	R^2*^	*P* ^*^	Correlation
p120-catenin membrane vs. β-catenin membrane	0.15	<0.01	positive
vs. β-catenin cytoplasm	0.07	0.03	inverse
p120-catenin cytoplasm vs. β-catenin membrane	0.13	<0.01	inverse
vs. β-catenin cytoplasm	0.15	<0.01	positive
**Invasive front**			
Parameter	R^2*^	*P* ^*^	Correlation
p120-catenin membrane vs. β-catenin membrane	0.33	<0.01	positive
vs. E-cadherin membrane	0.60	<0.01	positive
β-catenin membrane vs. E-cadherin membrane	0.29	<0.01	positive

* Regression analysis

### Catenin class switch and reduction under stimulation

**Figure 5:**
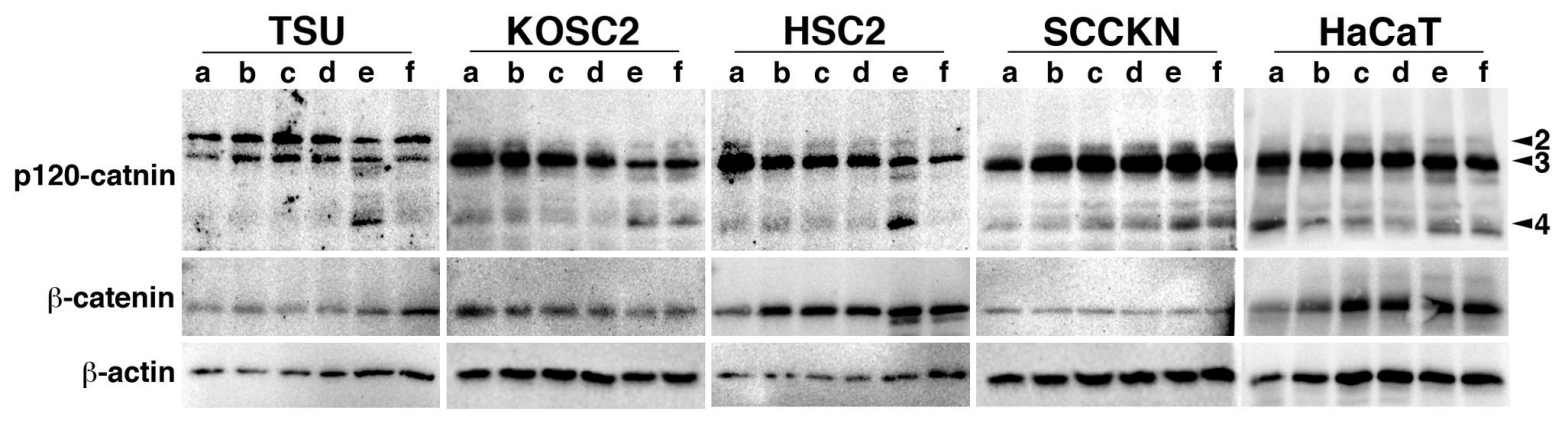
Effects of TGF-β, EGF and TNF-α on expression of catenins. Oral carcinoma cells (TSU, KOSC2, HSC2, SCCKN) and HaCaT cells were treated with TGF-β (lane b), TNF-α (lane c), EGF (lane d), TGF-β/TNF-α (lane e) or TGF-β/EGF (lane f), and subjected to immunoblot for p120-catenin and β-catenin. PBS was used to treat cells as a control (lane a). β-actin was used for the internal control.

Carcinoma cells and HaCaT cells were treated with tumor stimulus, TGF-β, TNF-α or EGF. These stimuli did not apparently affect the expression of p120-catenin isoforms (Figure 5). However, co-treatment of TGF-β with TNF-α or EGF reduced isoform 3, and produced a protein band at the same position as isoform 4. β-catenin was not affected by these treatments except for HSC2 cells in which the co-treatment partially degraded β-catenin. In fact, the densitometric analysis confirmed that the reduction in total amount of p120-catnin isoforms by TGF-β + EGF treatment (0.534 ± 0.389, mean ± S.D.; *P* = 0.0279) and TGF-β + TNF-α treatment (0.694 ± 0.326; *P* = 0.0688). β-catenin expression was not affect by the treatments (TGF-β + EGF, 0.867 ± 0.290, *P* = 0.3323; TGF-β + TNF-α, 0.842 ± 0.267, *P* = 0.2220). Single treatment of cells with TGF-β, TNF-α or EGF did not affect their expression (data not shown).

## Discussion

Catenins stabilize adherence junctions by binding to cadherins and to cytoskeletons. E-cadherin-mediated cell-cell adhesion regulates proliferation and differentiation of epithelial cells [18,19] and suppresses carcinoma development [20]. Carcinoma cell-cell adhesion of the epithelial origin is largely dependent on E-cadherin expression, and the reduction and loss of E-cadherin prompts aggressive features of carcinoma cells including migration, proliferation and degradation of extracellular matrix proteins [3]. In the current study, we showed that membrane staining of p120-catenin and β-catenin was rapidly decreased at the invasive front of aggressive oral carcinomas with loss of E-cadherin expression.

E-cadherin expression is inactivated by the transcriptional repressor binding and promoter hypermethylation in oral carcinomas [21,22]. In addition to the transcriptional level, it is negatively regulated by protein degradation and shedding [3]. The p120-catenin conditional knockout mouse exhibits hyperproliferation of skin keratinocytes with loss of E-cadherin protein and spontaneously develops invasive oral carcinomas [23,24]. Patients with advanced carcinomas frequently lose p120-catenin expression or mis-localize it in the cytoplasm and/or nucleus [24–27]. We confirmed that the loss of membrane expression was significant in the dedifferentiated and invasive carcinomas. However, the cytoplasmic/nuclear staining was limited and did not correlate with the clinicopathological parameters. The cluster analysis divided carcinomas with the membrane staining into p120-catenin^high^/E-cadherin^high^ and p120-catenin^low^/E-cadherin^low^ groups, suggesting that oral carcinoma cells at the invasive front concomitantly lose p120-catenin and E-cadherin expression. Loss of p120-catenin initiates E-cadherin protein degradation [5,28,29], but E-cadherin reduction and loss does not affect p120-catenin expression [5]. Loss of p120-catenin expression *per se* may define E-cadherin-mediated adhesion at the protein level independent of the transcriptional control in aggressive oral carcinomas. Although the mechanism for p120-catenin down-regulation in carcinoma tissues is not defined clearly, it is transcriptionally inactivated by ZEB2, an E-cadherin repressor that is predominantly expressed at the invasive front of oral carcinomas with worse prognosis [21,30]. The miR-197 that targets p120-catenin mRNA is expressed in oral carcinomas [31,32]. Unveiling the inhibitory mechanism for p120-catenin expression will contribute to understand the regulation of E-cadherin-mediated carcinoma cell-cell adhesion.

Among p120-catenin isoforms, this study confirmed the predominant expression of isoform 3 in oral carcinoma cells and normal keratinocytes. Since previous studies suggested the involvement of isoform shift from 3 to 1 in carcinoma progression [16,33], we treated cells with tumor stimulus (TGF-β, EGF and TNF-α) that facilitate the progression [34–36] and examined the shift. Unexpectedly, carcinoma cells did not show the shift. However, co-stimulation of TGF-β with TNF-α or EGF increased the isoform 4 or partially degraded them at the size of isoform 4, and reduced total amount of the isoform expression. In lung squamous cell carcinomas, loss of p120 catenin expression associates with tumor progression and the patient prognosis worsen regardless of the shift on the mRNA [37]. Since antibodies that can distinguish them are not available, we did not consider the isoform shift in carcinoma tissues and its pathological role in this study. However, the fact that the p120-catenin membrane staining was significantly decreased in advanced carcinomas and that the p120-catenin cytoplasmic staining was limited and did not associate with E-cadherin staining emphasizes again the impact of loss of p120-catenin expression, but not the subcellular localization and the isoform shift, on the carcinoma progression and loss of E-cadherin expression.

As in a number of previous studies, β-catenin staining at the cell membrane was decreased in parallel with carcinoma dedifferentiation and diffuse invasion, but the cytoplasmic/nuclear staining was detected in almost half of carcinoma cells. β-catenin did not alter its expression under TGF-β, EGF and TNF-α treatment that decreased p120-catenin, suggesting that the mechanism for β-catenin expression is largely different from p120-catenin. β-catenin localizes at the cytoplasm and nucleus in parallel with WNT expression in oral carcinoma cells at the invasive front [38]. In addition, β-catenin-mediated WNT signaling directly inactivates E-cadherin expression and up-regulates the E-cadherin repressors [39–41]. Multi-faceted events during carcinoma progression are activated by WNTs [42]. Although the cytoplasmic expression of p120-catenin enhances WNT signaling in an *in vitro* condition [8], it was limited in a small number of carcinoma cells at the invasive front. Loss of p120-catenin expression and the cytoplasmic/nuclear expression of β-catenin suggest the synergistic or stepwise regulation for loss of E-cadherin expression and what follows.

In normal oral epithelium, p120-catenin expression was weak and strictly restricted at the cell membrane of basal cells. The weak expression polarizes the basal cells to secrete basement membrane proteins to the underlying tissue [43], and genes involving the integrity of basal cell-types are frequently over-expressed in oral carcinomas [44,45]. In contrast to p120-catenin, β-catenin was diffusely detected at the cytoplasm of basal cells in a high frequency. Stratified squamous epithelial stem cells reside at the basal cell layer [46]. Since WNT signaling is indispensable for the development and maintenance of epithelial stem cells [47], the cytoplasmic localization of β-catenin and the membrane-restricted weak expression of p120-catenin at the basal cells strengthen the understanding that catenins play a distinct role in oral epithelium physiology.

E-cadherin expression is one of prime determinant for the carcinoma progression of epithelial origin [2] and it is regulated at the gene and protein levels [3]. Catenins play a decisive role in protein regulation. This study demonstrates that the immunohistochemical detection of p120-catenin and β-catenin at the membrane is an important indicator of carcinoma progression. In contrast to degradation of E-cadherin at the cell membrane by loss of p120-catenin expression [5,28,29], β-catenin gene targeting did not result in degradation [48]. Therefore, loss of p120-catenin expression may accelerate the progression by initiation of E-cadherin protein degradation and cytoplasmic/nuclear localization of β-catenin may strengthen the disease progression in synergistic and/or stepwise mechanisms.

## Materials and Methods

### Patient population

A total of 67 individual oral carcinomas were taken at Kanazawa University Hospital from biopsies or surgeries from 1988 to 2003. The median age of the study patients was 63.7-yrs (range, 37-93-yrs) at the time of diagnosis. The details of the pretreatment clinical and pathologic characteristics were summarized in Table S6. Histologic grading and staging were assessed according to the International Union Against Cancer (UICC) tumor-node-metastases classification. The mode of invasion classified carcinomas according to their histologic characters: grade 1, well-defined borderline; grade 2, cords, less marked borderline; grade3, groups of cells, no distinct borderline; grade 4C, diffuse invasion, cord-like type; and grade 4D, diffuse invasion, widespread type [49]. Normal oral epithelium was obtained from carcinoma-free patients. All tissues were obtained with the written consent of the patient and with approval by the institutional review boards of Kanazawa University and Nippon Dental University.

### Cell lines and cell treatments

Immortalized human oral carcinoma cell lines (TSU, HSC2, KOSC2, KOSC3, SCCKN, OSC19, and Ca9.22) were used [15]. Cells were maintained in 10% fetal bovine serum-containing DMEM or RPMI1640 medium (Sigma-Aldrich, St. Louis, MO) in a 5% CO_2_ incubator. Immortalized normal keratinocyte cell line, HaCaT [15,50], was maintained in 10% fetal bovine serum-containing DMEM. All cells were cultured until 80-90% confluency was obtained.

### Immunohistochemistry

Unstained formalin-fixed and paraffin-embedded sections of oral carcinomas and normal epithelium were treated with microwave (500 W) in 0.01 M sodium citrate buffer, pH 6.0, and incubated with mouse antibodies against p120-catenin (clone 98/pp120, BD Biosciences, Heidelberg, Germany) or β-catenin (clone 14, BD Biosciences) followed by biotinylated secondary antibodies (DAKO, Glostrup, Denmark). After treatment with avidin-biotin complexes (Vector Laboratories, Burlingame, CA), the color was developed with 3,3’-diaminobenzidine tetrahydrochloride. The percentage of carcinoma cells stained at the membrane or cytoplasm were calculated at least 3,000 cells for each section randomly selected. We determined that carcinoma cells with the strong circumferential membranous staining were membrane-positive and with strong cytoplasmic granular staining were cytoplasm-positive. The percentage of carcinoma cells stained membrane and cytoplasm was independently determined according to previous studies [15,51–54]. To clarify the specificity of the staining, sections were reacted with non-immune mouse IgG instead of primary antibodies.

### Immunoblot

The MB proteins and MNB proteins were isolated from oral carcinoma cells and HaCaT cells by the ProteoExtract Native Membrane Protein Extraction Kit (EMD Chemicals, Philadelphia, PA). After size-fractionation of proteins on SDS-polyacrylamid gels under the reduction (2 µg/lane) and electrotransferring to PDVF membranes, the membranes were probed with anti-p120-catenin or -β-catenin antibodies. Anti-Na/K ATPase (Abcam, Tokyo, Japan) and -β-actin (Sigma-Aldrich) antibodies were used for the positive controls of MB and MNB fractions, respectively. To determine the p120-catenin isoform shift by immunoblotting, cells were incubated in serum-free medium overnight, treated with human TGF-β (10 ng/ml; PeproTech, Rocky Hill, NJ), EGF (100 ng/ml; PeproTech) and TNF-α (10 ng/ml; Sigma-Aldrich) for 48 h, and harvested the hole-cell lysates in SDS-polyacrylamide gel sample buffer. The membranes were put on a flatbed scanner, and intensity of the bands was compared using the ImageJ 1.46r [55].

### Statistical analysis

Association of percentage of catenin-positive carcinoma cells and the clinicopathological parameters were analyzed by Welch’s ANOVA, regression analysis or Mann-Whitney *U* test using JMP 7.0.1 (SAS Institute Inc., Cary, NC). To analyze the percentage of catenin-positive cells and E-cadherin-positive cells at the invasive front (*n* = 39), the raw data for E-cadherin-positive cells were quoted from our previous study published in PLoS ONE [15] and subjected to regression and cluster analyses. Expression of total p120-catenin isoforms and β-catenin on the immunoblot membranes was standardized by that of β-actin and analyzed by student’s t-test.

## Supporting Information

Table S1 **Percentage of p120-catenin-membrane positive carcinoma cells and clinicopathological implications.**
(DOC)

Table S2 **Percentage of p120-catenin-cytoplasm stained carcinoma cells and clinicopathological implications.**
(DOC)

Table S3 **Percentage of β-catenin-membrane positive carcinoma cells and clinicopathological implications.**
(DOC)

Table S4 **Percentage of β-catenin-cytoplasm stained carcinoma cells and clinicopathological implications.**
(DOC)

Table S5 **Correlation of percentage of catenin and cadherin positive carcinoma cells.**
(DOC)

Table S6 **Clinicopathological parameters of 67 primary oral carcinomas.**
(DOC)
