# Supplementary material for: Concomitant Loss of p120-Catenin and β-Catenin Membrane Expression and Oral Carcinoma Progression with E-Cadherin Reduction
Source: PLoS One. 2013 Aug 6;8(8):e69777. doi: 10.1371/journal.pone.0069777 (PMC3735538; doi:10.1371/journal.pone.0069777)
Supplement: Table S1 — Percentage of p120-catenin-membrane positive carcinoma cells and clinicopathological implications. (DOC) [file pone.0069777.s001.doc]

**Table S1.** Percentage of p120-catenin-membrane positive carcinoma cells and clinicopathological implications.

Parameters Center Invasive front

mean ± SD *P*^†^ Mean ± SD *P*^†^

Age 0.16 0.27

≤ 65 yrs 71.59 ± 24.35 27.73 ± 31.54

> 65 yrs 61.83 ± 29.44 19.59 ± 27.40

Sex 0.51 0.78

female 64.76 ± 27.90 26.04 ± 32.65

male 69.23 ± 26.08 23.92 ± 28.55

T stage^‡^ 0.05 0.09

T1 77.71 ± 18.60 38.88 ± 38.14

T2 60.06 ± 27.69 18.90 ± 23.07

T3 55.71 ± 39.42 8.86 ± 18.51

T4 78.41 ± 17.70 29.42 ± 33.07

N stage^‡^ 0.61 0.36

N0 70.86 ± 23.87 28.53 ± 31.99

N1 63.35 ± 26.56 16.94 ± 23.89

N2 65.00 ± 36.33 24.50 ± 32.95

N3 0.00 0.00

Clinical stage^‡^ 0.25 0.18

stage 1 75.94 ± 19.76 39.41 ± 37.88

stage 2 65.29 ± 22.42 17.05 ± 22.30

stage 3 56.77 ± 33.07 16.31 ± 23.91

stage 4 69.63 ± 31.50 26.38 ± 30.74

Histological differentiation < 0.01 0.01

well 76.20 ± 17.80 36.07 ± 31.78

moderately 73.33 ± 20.75 19.75 ± 25.70

poorly 36.00 ± 31.75 8.15 ± 24.68

Mode of invasion^*^ 0.11 < 0.01

grade 1 76.70 ± 17.04 47.30 ± 33.49

grade 2 78.00 ± 24.86 21.67 ± 28.93

grade 3 69.79 ± 21.76 27.75 ± 30.12

grade 4C 59.38 ± 38.72 7.75 ± 17.29

grade 4D 35.71 ± 27.40 0.00 ± 0.00

^†^ Probability of statistical difference (*P*) was analyzed by Welch’s ANOVA.

^‡^ Patients were categorized by tumor size (T stage), lymph node metastasis (N stage) and clinical stages according to the International Union against Cancer (UICC) WHO grading system.

^*^ Patients were categorized by mode of invasion.
