# Supplementary material for: Concomitant Loss of p120-Catenin and β-Catenin Membrane Expression and Oral Carcinoma Progression with E-Cadherin Reduction
Source: PLoS One. 2013 Aug 6;8(8):e69777. doi: 10.1371/journal.pone.0069777 (PMC3735538; doi:10.1371/journal.pone.0069777)
Supplement: Table S2 — Percentage of p120-catenin-cytoplasm stained carcinoma cells and clinicopathological implications. (DOC) [file pone.0069777.s002.doc]

**Table S2.** Percentage of p120-catenin-cytoplasm stained carcinoma cells and clinicopathological implications.

Parameters Center Invasive front

Mean ± SD *P*^†^ Mean ± SD *P*^†^

Age 0.46 0.79

≤ 65 yrs 16.87 ± 21.30 17.95 ± 26.87

> 65 yrs 21.21 ± 25.75 19.62 ± 24.39

Sex 0.43 0.54

female 21.32 ± 22.15 16.79 ± 24.62

male 16.82 ± 23.87 20.69 ± 26.51

T stage^‡^ 0.06 0.64

T1 19.12 ± 23.58 27.47 ± 36.32

T2 24.23 ± 26.90 17.29 ± 20.49

T3 8.14 ± 14.52 15.00 ± 29.01

T4 10.00 ± 8.08 14.08 ± 16.13

N stage^‡,*^ < 0.01 0.15

N0 16.91 ± 23.85 21.74 ± 27.98

N1 24.35 ± 18.93 16.29 ± 23.61

N2 5.33 ± 4.08 10.83 ± 8.01

N3 80.00 0.00

Clinical stage^‡^ 0.60 0.95

stage 1 16.00 ± 15.00 22.24 ± 31.26

stage 2 18.05 ± 26.38 17.33 ± 20.89

stage 3 25.69 ± 21.68 19.54 ± 26.24

stage 4 16.75 ± 27.37 17.56 ± 26.36

Histological differentiation 0.72 0.63

well 16.70 ± 20.07 20.70 ± 25.94

moderately 18.04 ± 21.82 20.08 ± 27.27

poorly 24.54 ± 31.64 13.38 ± 22.69

Mode of invasion^§^ 0.49 0.99

grade 1 18.90 ± 28.59 20.50 ± 26.62

grade 2 11.83 ± 15.67 21.67 ± 32.49

grade 3 17.18 ± 20.49 19.07 ± 24.56

grade 4C 32.38 ± 37.38 16.25 ± 19.72

grade 4D 17.29 ± 13.50 13.71 ± 29.49

^†^ Probability of statistical difference (*P*) was analyzed by Welch’s ANOVA.

^‡^ Patients were categorized by tumor size (T stage), lymph node metastasis (N stage) and clinical stages according to the International Union against Cancer (UICC) WHO grading system.

^*^ A significant difference at the center may be spurious because N2 (5.33 ± 4.08) was lower than others (N0, 16.91 ± 23.85; N1, 24.35 ± 18.93; N3, 80.00).

^§^ Patients were categorized by mode of invasion.
