# Supplementary material for: Concomitant Loss of p120-Catenin and β-Catenin Membrane Expression and Oral Carcinoma Progression with E-Cadherin Reduction
Source: PLoS One. 2013 Aug 6;8(8):e69777. doi: 10.1371/journal.pone.0069777 (PMC3735538; doi:10.1371/journal.pone.0069777)
Supplement: Table S3 — Percentage of β-catenin-membrane positive carcinoma cells and clinicopathological implications. (DOC) [file pone.0069777.s003.doc]

**Table S3.** Percentage of β-catenin-membrane positive carcinoma cells and clinicopathological implications.

Parameters Center Invasive front

Mean ± SD *P*^†^ Mean ± SD *P*^†^

Age 0.66 0.01

≤ 65 yrs 65.95 ± 28.02 29.00 ± 28.83

> 65 yrs 62.79 ± 29.97 14.59 ± 15.60

Sex 0.68 0.62

female 66.46 ± 22.52 25.18 ± 24.12

male 63.69 ± 32.49 22.03 ± 26.29

T stage^‡^ 0.67 0.92

T1 70.76 ± 25.65 25.41 ± 26.31

T2 62.45 ± 26.50 21.90 ± 24.52

T3 71.14 ± 32.50 19.29 ± 29.38

T4 59.08 ± 36.41 26.50 ± 26.07

N stage^‡^ 0.11 0.41

N0 71.53 ± 23.24 24.09 ± 23.73

N1 52.94 ± 28.60 17.47 ± 23.41

N2 61.00 ± 44.55 38.50 ± 37.94

N3 3.00 0.00

Clinical stage^‡^ 0.52 0.55

stage 1 69.06 ± 28.94 27.65 ± 27.31

stage 2 70.00 ± 23.16 22.81 ± 22.99

stage 3 60.31 ± 24.43 15.08 ± 23.19

stage 4 57.31 ± 37.08 26.19 ± 28.09

Histological differentiation 0.01 0.01

well 64.30 ± 26.65 22.13 ± 21.07

moderately 76.58 ± 20.70 32.63 ± 30.40

poorly 44.46 ± 26.65 9.00 ± 16.07

Mode of invasion* 0.91 0.03

grade 1 71.50 ± 14.10 23.80 ± 22.95

grade 2 68.17 ± 23.61 26.25 ± 21.47

grade 3 63.46 ± 33.26 27.75 ± 27.54

grade 4C 61.00 ± 35.86 16.88 ± 28.36

grade 4D 64.29 ± 23.64 4.42 ± 5.71

^†^ Probability of statistical difference (*P*) was analyzed by Welch’s ANOVA.

^‡^ Patients were categorized by tumor size (T stage), lymph node metastasis (N stage) and clinical stages according to the International Union against Cancer (UICC) WHO grading system.

^*^ Patients were categorized by mode of invasion.
