# Supplementary material for: Concomitant Loss of p120-Catenin and β-Catenin Membrane Expression and Oral Carcinoma Progression with E-Cadherin Reduction
Source: PLoS One. 2013 Aug 6;8(8):e69777. doi: 10.1371/journal.pone.0069777 (PMC3735538; doi:10.1371/journal.pone.0069777)
Supplement: Table S4 — Percentage of β-catenin-cytoplasm stained carcinoma cells and clinicopathological implications. (DOC) [file pone.0069777.s004.doc]

**Table S4.** Percentage of β-catenin-cytoplasm stained carcinoma cells and clinicopathological implications.

Parameter Center Invasive front

Mean ± SD *P*^†^ Mean ± SD *P*^†^

Age 0.88 0.71

≤ 65 yrs 24.59 ± 25.74 49.27 ± 30.36

> 65 yrs 23.62 ± 24.17 46.38 ± 32.69

Sex 0.13 0.13

female 18.75 ± 18.54 40.75 ± 29.31

male 27.59 ± 28.98 52.38 ± 31.98

T stage^‡,*^ 0.02 0.07

T1 22.00 ± 29.71 57.47 ± 31.33

T2 28.77 ± 26.55 47.42 ± 31.40

T3 10.71 ± 7.30 23.71 ± 23.65

T4 21.67 ± 20.89 47.58 ± 30.08

N stage^‡,*^ 0.03 0.58

N0 20.72 ± 22.74 47.23 ± 30.59

N1 33.18 ± 28.25 50.24 ± 31.90

N2 9.50 ± 10.88 33.83 ± 31.83

N3 89.00 96.00

Clinical stage^‡^ 0.06 0.76

stage 1 19.71 ± 26.60 53.88 ± 32.74

stage 2 20.52 ± 18.07 47.95 ± 28.77

stage 3 33.38 ± 28.98 40.31 ± 34,73

stage 4 25.06 ± 29.22 46.06 ± 31.28

Histological differentiation 0.41 0.28

well 22.10 ± 25.36 44.50 ± 30.23

moderately 20.96 ± 22.79 43.79 ± 29.76

poorly 33.46 ± 29.41 61.38 ± 34.49

Mode of invasion^*,§^ < 0.01 0.03

grade 1 19.00 ± 14.19 52.90 ± 24.28

grade 2 31.33 ± 27.84 55.42 ± 28.29

grade 3 23.61 ± 26.30 47.68 ± 31.36

grade 4C 32.13 ± 36.88 51.00 ± 40.49

grade 4D 15.29 ± 14.43 33.00 ± 32.26

^†^ Probability of statistical difference (*P*) was analyzed by Welch’s ANOVA.

^‡^ Patients were categorized by tumor size (T stage), lymph node metastasis (N stage) and clinical stages according to the International Union against Cancer (UICC) WHO grading system.

^*^ Significant difference may be spurious because the non-advanced vs. advanced-carcinomas (T-stage, T1 vs. T2-4; N-stage, N0 vs. N1-3; mode of invasion, grade 1/2 vs. 4C/4D) were equivalent.

^§^ Patients were categorized by mode of invasion.
