# Supplementary material for: Concomitant Loss of p120-Catenin and β-Catenin Membrane Expression and Oral Carcinoma Progression with E-Cadherin Reduction
Source: PLoS One. 2013 Aug 6;8(8):e69777. doi: 10.1371/journal.pone.0069777 (PMC3735538; doi:10.1371/journal.pone.0069777)
Supplement: Table S5 — Correlation of percentage of catenin and cadherin positive carcinoma cells. (DOC) [file pone.0069777.s005.doc]

**Table S5.** Correlation of percentage of catenin and cadherin positive carcinoma cells.

Parameter R^2 †^ *P* ^†^ Correlation

Center

p120-catenin membrane vs. β-catenin membrane 0.15 0.001 positive

vs. β-catenin cytoplasm 0.07 0.028 inverse

p120-catenin cytoplasm vs. β-catenin membrane 0.13 0.003 inverse

vs. β-catenin cytoplasm 0.15 0.001 positive

Invasive front

p120-catenin membrane vs. β-catenin membrane 0.33 < 0.001 positive

vs. β-catenin cytoplasm 0.05 0.073 inverse

vs. E-cadherin membrane 0.06 < 0.001 positive

vs. E-cadherin cytoplasm 0.16 0.011 positive

p120-catenin cytoplasm vs. β-catenin membrane 0.01 0.324 inverse

vs. β-catenin cytoplasm 0.03 0.201 positive

vs. E-cadherin membrane 0.02 0.429 positive

vs. E-cadherin cytoplasm 0.01 0.492 positive

β-catenin membrane vs. E-cadherin membrane 0.29 0.001 positive

vs. E-cadherin cytoplasm 0.07 0.114 positive

β-catenin cytoplasm vs. E-cadherin membrane 0.07 0.102 inverse

vs. E-cadherin cytoplasm 0.05 0.192 inverse

^†^Probability of statistical difference (*P*) and R^2^ were analyzed by regression analysis.
