# Supplementary material for: Concomitant Loss of p120-Catenin and β-Catenin Membrane Expression and Oral Carcinoma Progression with E-Cadherin Reduction
Source: PLoS One. 2013 Aug 6;8(8):e69777. doi: 10.1371/journal.pone.0069777 (PMC3735538; doi:10.1371/journal.pone.0069777)
Supplement: Table S6 — Clinicopathological parameters of 67 primary oral carcinomas. (DOC) [file pone.0069777.s006.doc]

**Table S6.** Clinicopathological parameters of 67 primary oral carcinomas.

Parameter *n* Parameter *n*

Age M stage^‡^

≤ 65 yrs 37 M0 64

> 65 yrs 30 M1 3

Sex Clinical stage^‡^

female 28 stage 1 17

male 39 stage 2 21

T stage^‡^ stage 3 13

T1 17 stage 4 16

T2 31 Histological differentiation

T3 7 well 30

T4 12 moderately 24

N stage^‡^ poorly 13

N0 43 Mode of invasion ^*^

N1 17 grade 1 10

N2 6 grade 2 12

N3 1 grade 3 28

grade 4C 8

grade 4D 7

^‡^ Patients were categorized by tumor size (T stage), lymph node metastasis (N stage) and clinical stages according to the International Union against Cancer (UICC) WHO grading system.

^*^ Patients were categorized by mode of invasion.
